# Clinical and genetic characteristics of Chinese patients with congenital cranial dysinnervation disorders

**DOI:** 10.1186/s13023-022-02582-5

**Published:** 2022-12-09

**Authors:** Hongyan Jia, Qian Ma, Yi Liang, Dan Wang, Qinglin Chang, Bo Zhao, Zongrui Zhang, Jing Liang, Jing Song, Yidi Wang, Ranran Zhang, Zhanhan Tu, Yonghong Jiao

**Affiliations:** 1 Beijing Tongren Eye Center, Beijing Tongren Hospital, Capital Medical University, Beijing, 100730 China; 2 Beijing Ophthalmology and Visual Science Key Lab, Beijing, 100730 China; 3 Department of Radiology, Beijing Tongren Hospital, Capital Medical University, Beijing, 100730 China; 4 Department of Neuroscience, Psychology and Behaviour, Ulverscroft Eye Unit, University of Leicester, Robert Kilpatrick Clinical Sciences Building, Leicester Royal Infirmary, Leicester, LE2 7LX UK

**Keywords:** Congenital cranial dysinnervation disorders, Magnetic resonance imaging, Whole exome sequencing, Phenotypic and genotypic characteristics, Genetics

## Abstract

**Background:**

Congenital cranial dysinnervation disorders (CCDDs) are a group of diseases with high clinical and genetic heterogeneity. Clinical examinations combined with Magnetic resonance imaging (MRI) and whole exome sequencing (WES) were performed to reveal the phenotypic and genotypic characteristics in a cohort of Chinese CCDDs patients.

**Results:**

A total of 122 CCDDs patients from 96 families were enrolled. All patients showed restrictive eye movements, and 46 patients from 46 families (47.9%, 46/96) were accompanied by multiple congenital malformations. Multi-positional high-resolution MRI was performed in 94 patients from 88 families, of which, all patients had hypoplasia of the cranial nerves except HGPPS patients and 15 patients from 15 families (17.0%,15/88) were accompanied by other craniocerebral malformations. WES was performed in 122 CCDDs patients. Ten pathogenic variants were detected in *KIF21A*, *TUBB3*, *and CHN1* genes in 43 families. Three variants were unreported, including *KIF21A* (c.1064T > C, p.F355S), *TUBB3* (c.232T > A, p.S78T) and *CHN1* (c.650A > G, p.H217R). Of the 43 probands harboring pathogenic variants, 42 were diagnosed with Congenital Fibrosis of Extraocular Muscles (CFEOM) and one was Duane Retraction Syndrome (DRS). No definite pathogenic variants in known candidate genes of CCDDs were found in sporadic DRS, Möbius Syndrome (MBS) and Horizontal Gaze Palsy with Progressive Scoliosis (HGPPS) patients. The CFEOM patients harboring R380C, E410K and R262H variants in *TUBB3* gene and F355S variant in *KIF21A* gene exhibited syndromic phenotypes.

**Conclusions:**

This study broadened the phenotypic and genotypic spectrums of CCDDs, and it was the largest clinical and genetic investigation for CCDDs patients from China. *KIF21A* and *TUBB3* were the common pathogenic genes in Chinese CFEOM. MRI coupled with WES can provide a supportive diagnosis in patients with clinically suspected CCDDs.

**Supplementary Information:**

The online version contains supplementary material available at 10.1186/s13023-022-02582-5.

## Background

Congenital cranial dysinnervation disorders (CCDDs) are a group of congenital, non-progressive, sporadic or familial abnormalities of innervation of extraocular and cranial musculature with high clinical and genetic heterogeneity. The CCDDs can be resulted from hypoplasia or absence of one or more cranial nerves with primary or secondary muscle dysinnervation [1]. The disease spectrum of CCDDs encompasses Congenital Fibrosis of Extraocular Muscles (CFEOM), Duane Retraction Syndrome (DRS), Möbius Syndrome (MBS), Horizontal Gaze Palsy with Progressive Scoliosis (HGPPS) and Hereditary Congenital Facial Paresis (HCFP) etc. [2]. CCDDs can be accompanied by multiple congenital malformations, for instance, craniofacial malformations, musculoskeletal defects of limb and/or trunk, intellectual and behavioral impairments etc. [3, 4].

So far, causative variants have been identified in a dozen genes, including *KIF21A*, *TUBB3*, *TUBA1A*, *TUBB2B*, *TUBB6, PHOX2A*, *CHN1*, *MAFB*, *SALL4, HOXA1, HOXB1, ROBO3, PLXND1, REV3L, COL25A1* and *ECEL1* etc. [5–19]. Previous reports have established that at least a subset of these genes is critical to the differentiation and migration of the cranial motor neurons, patterning of brainstem, as well as the axon growth and guidance [2, 5–19].

The phenotypic spectrum of CCDDs is broad, and the phenotypes among different constituent diseases may be overlapping [3]. Hypoplasia of the cranial nerves is the most common characteristic of CCDDs. Magnetic resonance imaging (MRI) can display brain structure, cranial nerves and extraocular muscles in high resolution, so MRI can provide supporting evidence for clinical suspected CCDDs cases [20, 21]. Furthermore, whole exome sequencing (WES) can assist with genetic diagnosis and classification of CCDDs [3]. In this study, clinical examination, MRI and WES were used to reveal the clinical and genetic characteristics in a cohort of Chinese Han CCDDs patients.

## Results

### Clinical and MRI evaluation results of CCDDs

**Fig. 1:**
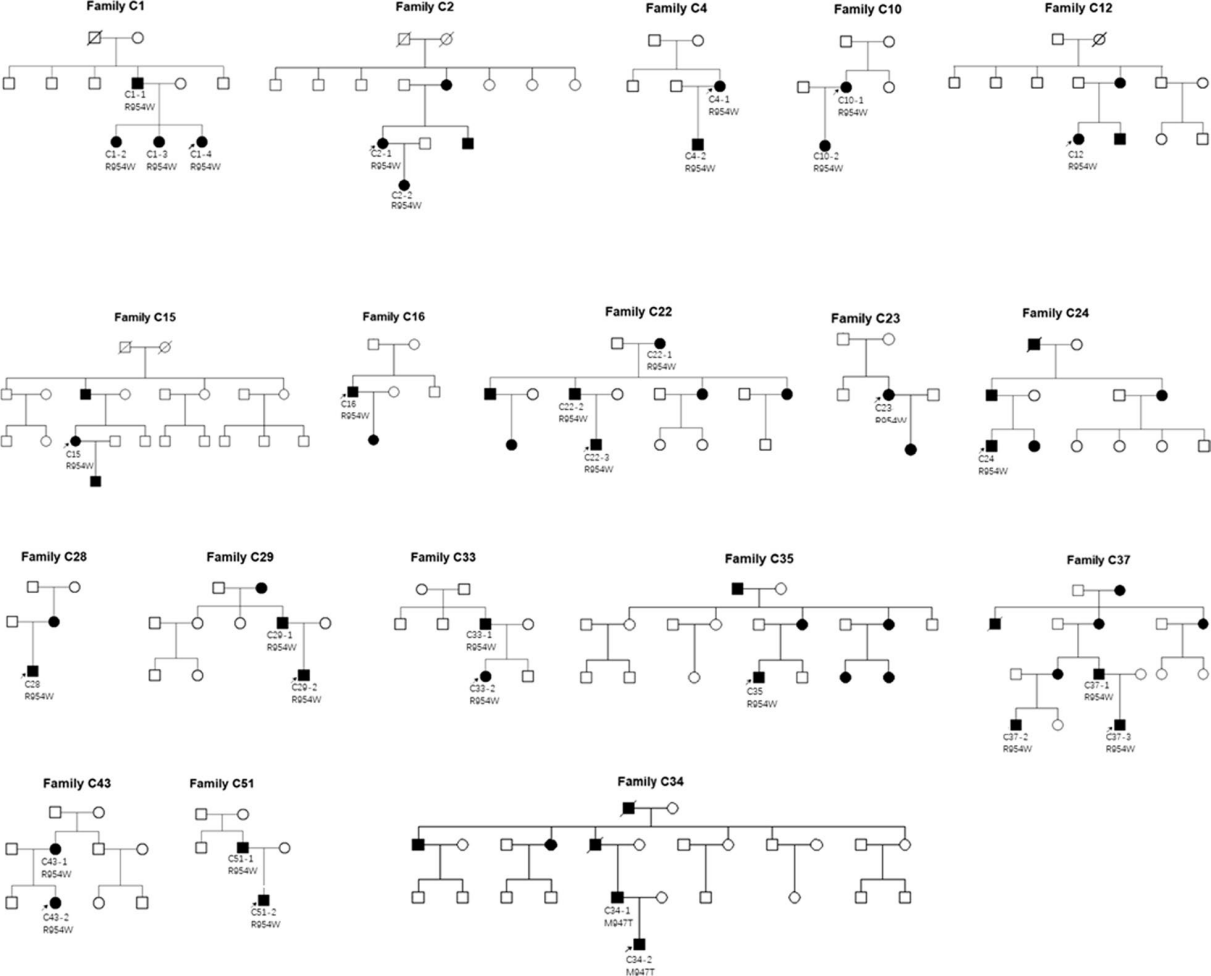
The family trees of familial forms CFEOM patients carrying *KIF21A* variants

**Fig. 2:**
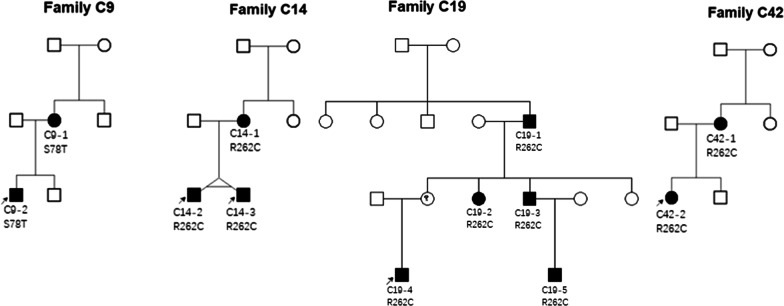
The family trees of familial forms CFEOM patients carrying *TUBB3* variants

A total of 122 Chinese self-reported Han patients from 96 not known to be related families with CCDDs were enrolled in the study (24 familial and 72 sporadic; age ranged from 5 months to 60 years); their demographic data and systemic features are provided in Table 1. The family trees of familial CFEOM patients were shown in Figs. 1 and 2. All 122 patients have different degrees of restriction of eye movements. Of them, 46 patients from 46 families (47.9%, 46/96) were accompanied by multiple congenital malformations, including facial weakness, joint constructure and psychomotor retardation etc.

**Table 1:** Demographics and systemic features of CCDDs patients

	CFEOM	DRS	MBS	HGPPS	Total
No. of patients/families	89/66	11/8	17/17	5/5	122/96
Sex					
Males	48/89 (53.9%)	6/11 (54.5%)	8/17 (47.1%)	3/5 (60%)	65/122 (53.3%)
Female	41/89 (46.1%)	5/11 (45.5%)	9/17 (52.9%)	2/5 (40%)	57/122 (46,7%)
No. of pedigrees	22	2	0	0	24
Sporadic cases	44	6	17	5	72
MRI (performed)					
No. of patients	64	10	16	4	94
Multiple malformations*	17	7	17	5	46
Craniocerebral malformation	9	1	1	4	15

*Multiple malformations contain craniocerebral malformation and other systemic malformations

94 patients from 88 families underwent MRI, of which, 90 patients exhibited hypoplasia of cranial nerves except HGPPS patients. Furthermore, fifteen patients from 15 families (17.0%, 15/88) were accompanied by other craniocerebral dysplasia, which affected commissural fiber, basal ganglia, thalamus, cerebral ventricle, brainstem and cerebellum etc. An additional table file shows the clinical data of all CCDDs patients in more detail (see Additional file 1).

### WES evaluation results of CCDDs

**Fig. 3:**
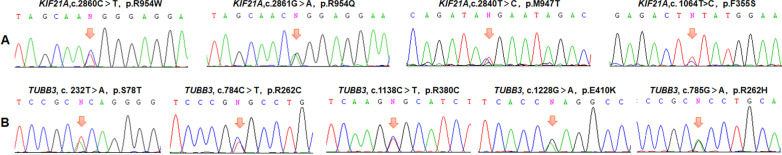
Genomic sequence chromatograms of CFEOM individuals with *KIF21A* and *TUBB3* variants. **A** Four heterozygous variants (R954W, R954Q, M947T and F355S) of *KIF21A* gene (red arrows). **B** Five heterozygous variants (S78T, R262C, R380C, E410K and R262H) of *TUBB3* gene (red arrows)

**Fig. 4:**
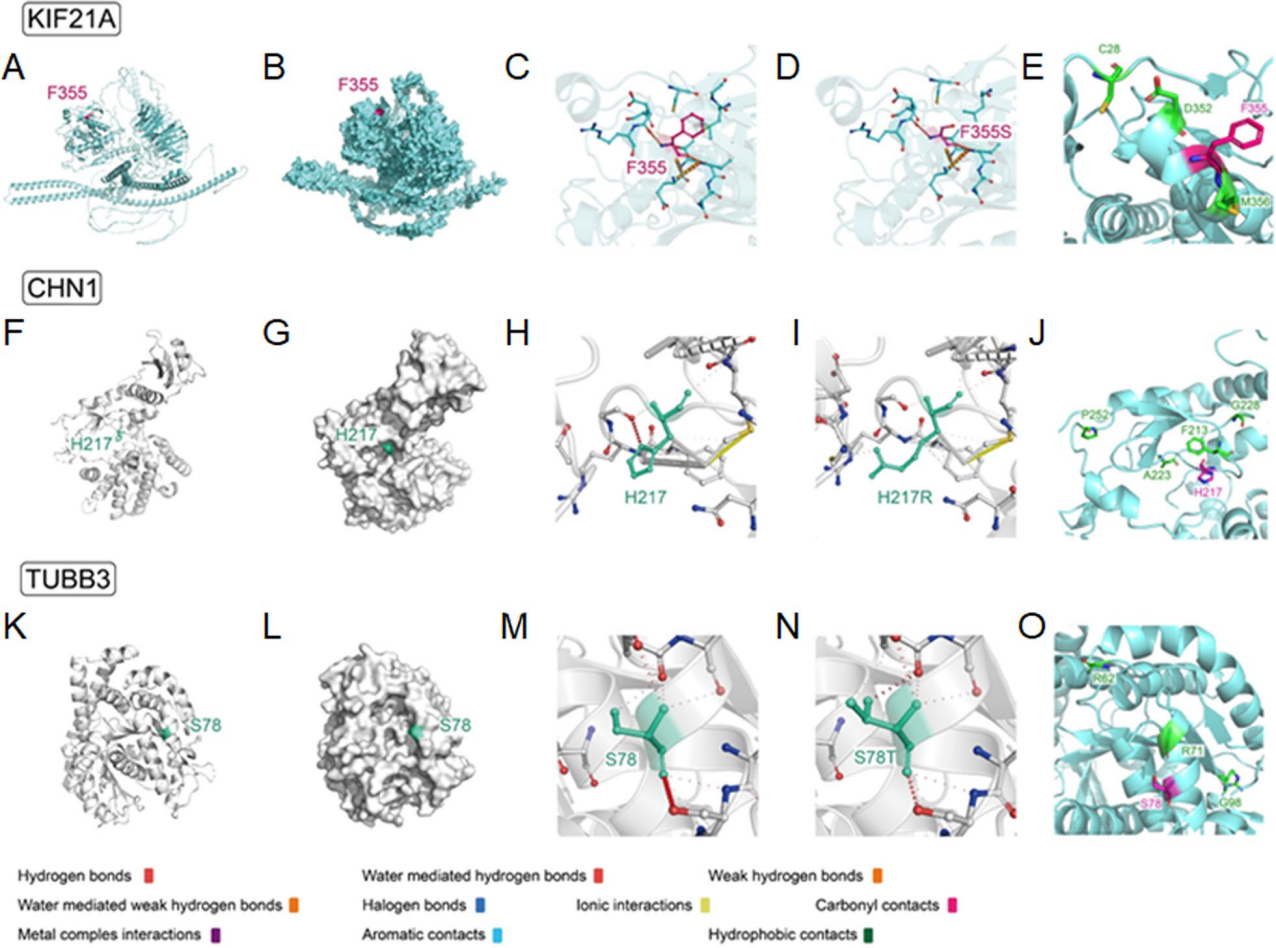
Spatial distribution of protein key sites and changes of amino acid interaction force before and after mutation. **A**, **B** KIF21A protein structure (cartoon/surface form) and spatial distribution of F355 residue. **C**, **D** Interaction force analysis of the KIF21A F355 and KIF21A F355S with the surrounding amino acid residues. **E** Showing positions of F355 and previously reported variants associated with CFEOM in the motor domain. **F**, **G** CHN1 protein structure (cartoon/surface form) and spatial distribution of H217 residue. **H**, **I** Interaction force analysis of the CHN1 H217 and CHN1 H217R with the surrounding amino acid residues. **J** Showing positions of H217 and other nearby previously reported variants in the same domain. **K**, **L** TUBB3 protein structure (cartoon/surface form) and spatial distribution of S78 residue. **M**, **N** Interaction force analysis of the TUBB3 S78 and TUBB3 S78T with the surrounding amino acid residues. **O** Showing positions of S78 and other nearby previously reported variants in the same domain

Using WES, we identified 10 pathogenic variants in the *KIF21A*, *TUBB3* and *CHN1* genes in 43 families (24 familial and 19 sporadic). Genomic sequence chromatograms of CFEOM individuals with *KIF21A* and *TUBB3* variants were shown in Fig. 3. Three variants were novel: *KIF21A* (c.1064T > C, p.F355S) (NM_001173464), *TUBB3* (c.232T > A, p.S78T) (NM_006086) and *CHN1* (c.650A > G, p.H217R) (NM_001371513), and they were not found in Chinese ethnically-matched databases, gnomAD, etc. (see Additional file 2). CNVs were not identified in known pathogenic genes of CCDDs. For three unreported variants, the 3D structure prediction and stability analysis of mutated protein were performed (Table 2 and Fig. 4).

**Table 2:** The stability analysis of mutations by different servers and software

Mutation (kcal/mol)	ΔΔG DynaMut	ΔΔG ENCoM	ΔΔG mCSM	ΔΔG SDM	ΔΔG DUET	ΔΔG FoldX
KIF21A F355S	− 1.974	− 0.998	− 1.709	− 1.400	− 1.717	3.006
	Destabilizing	Destabilizing	Destabilizing	Destabilizing	Destabilizing	Destabilizing
CHN1 H217R	− 0.395	0.021	− 1.270	− 0.390	− 1.108	0.204
	Destabilizing	Destabilizing	Destabilizing	Destabilizing	Destabilizing	Stabilizing
TUBB3 S78T	− 0.066	− 0.063	− 0.206	0.090	0.227	− 0.4751
	Destabilizing	Destabilizing	Destabilizing	Stabilizing	Stabilizing	Stabilizing

The protein spatial structure of KIF21A was predicted by SWISS-MODEL, and F355 is located in the possible protein binding pocket (Fig. 4A, B). Force analysis showed a weakened interaction force between the amino acid residues at F355 and the surrounding amino acid residues after the mutation (Fig. 4C, D). The free energy analysis indicated that the free energy of the whole structure changed greatly, and the stability of the protein structure decreased after the mutation (Table 2).

The CHN1 protein structure came from PDBID:3CXL, and H217 is located in the binding pocket of the spatial structure (Fig. 4F, G). Force analysis showed a weakened interaction force after the mutation (Fig. 4H, I). The free energy analysis indicated that the free energy changed greatly, and the stability of the protein structure decreased after the mutation, suggested that H217 is a key site to maintain the structural stability of CHN1 (Table 2).

The TUBB3 protein structure came from PDBID:7PJF, and S78 is located in the surface of spatial structure (Fig. 4K, L). No obvious change in interaction force was found after the mutation (Fig. 4M, N). No obvious change in the free energy was found after the mutation, indicated that the structural stability remains unchanged after mutation (Table 2).

Among the 43 patients harboring variants, 42 were diagnosed with CFEOM and one was familial DRS. No potential pathogenic variants in known candidate genes of CCDDs variant were found in sporadic DRS and MBS. For HGPPS, more than ten heterozygous variants were found in *ROBO3* gene, according to American College of Medical Genetics and Genomics (ACMG) standards, no variants were classified as pathogenic or likely pathogenic. 

### Genotype–phenotype results in CCDDs

#### CFEOM

The study enrolled 89 CFEOM cases from 66 families (22 familial and 44 sporadic), all patients showed ptosis, variable limited eye movements and restrictive strabismus. The overall mutation detection rate of CFEOM was 63.6% (42/66), and mutations were detected in 100% familial CFEOM cases. Nine mutations (7 previously reported and 2 novel) in the *KIF21A* (73.8%, 31/42) and *TUBB3* (26.2%, 11/42) genes were identified in 42 probands (22 familial and 20 sporadic).

#### KIF21A

**Fig. 5:**
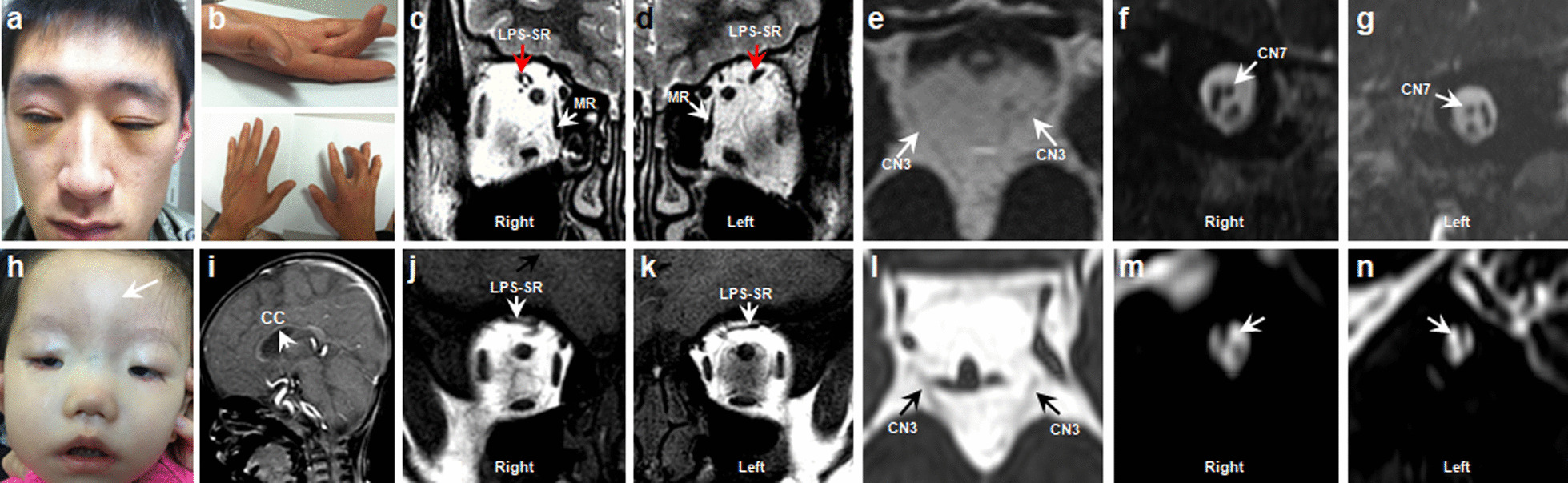
Clinical spectrum and MRI findings of patient C3 (**a**–**g**) and C46 (**h**–**n**). **a**, **b** Photographs of patient C3 harboring *KIF21A* R954W shows congenital bilateral ptosis (after correction of blepharoptosis) and joint contracture of right middle finger. **c**–**g** MRI findings of patient C3 (The MRI was taken at the age of 16). Atrophy of bilateral levator palpebrae superioris-superior rectus (LPS-SR) and bilateral medial rectus (MR), hypoplasia of bilateral oculomotor nerves (CN3), and bilateral facial nerves (CN7) are normal (**f**, **g**). **h** Facial photograph of patient C46 harboring *KIF21A* F355S shows congenital bilateral ptosis, facial weakness and bony prominence (arrow). **i**–**n** MRI findings of patient C3 (The MRI was taken at the age of 1y). Hypoplasia of corpus callosum (CC), LPS-SR, CN3 and absence of CN7(**m**–**n**, arrow)

Four variants (R954W, R954Q, M947T and F355S) in *KIF21A* gene were identified in 31 CFEOM families. The two most common variants were R954W (77.4%, 24/31) and R954Q (16.1%, 5/31), and the other two less common variants were M947T (3.2%, 1/31) and F355S (3.2%, 1/31). The c.1064T > C, p.F355S was an unreported de novo variant. We observed that almost all CFEOM patients harboring *KIF21A* variants showed similar phenotypes, that is isolated CFEOM, except for patients C3 (R954W) and C46 (F355S). The patient C3 was also accompanied by congenital distal joint contracture, and the patient C46 exhibited facial weakness, frontal bony prominence, delayed developmental milestones and thinning of corpus callosum in MRI (Fig. 5). Furthermore, we also observed that patients harboring the same *KIF21A* variants may also have different severity of CFEOM, even if they come from the same family.

#### TUBB3

Five variants (R262C, R262H, E410K, R380C and S78T) were identified in *TUBB3* gene in 11 CFEOM families. The two most common variants were R262C (54.5%, 6/11) and E410K (18.2%, 2/11), less commonly, R380C (9.1%, 1/11), R262H (9.1%, 1/11) and a novel variant S78T (9.1%, 1/11). Clinical and genetic findings of the 11 CFEOM probands with *TUBB3* variants were listed in Table 3.

**Table 3:** Clinical and genetic findings of the 11 CFEOM probands with *TUBB3* variants

Subject	C9-2	C5	C14-2	C19-4	C31	C42-2	C65	C6	C21	C32	C52
Gender/Age	M/3y	F/3y	M/5y	M/3y	F/14y	F/6y	F/3y	M/10y	M/11y	F/19y	M/3y
Family history	+	−	+	+	−	+	−	−	−	−	−
*Genetic findings*											
cDNA variantion	c.232T > A	c.784C > T	c.784C > T	c.784C > T	c.784C > T	c.784C > T	c.784C > T	c.1138C > T	c.1228C > T	c.1228C > T	c.785G > A
AA change	p. S78T	p. R262C	p. R262C	p. R262C	p. R262C	p. R262C	p. R262C	p. R380C	p. E410K	p. E410K	p. R262H
*Ophthalmological characteristics*											
Ptosis	R	B	L	B	B	B	B	B	B	B	R
Ocular alignment	ET, HoT	XT, HoT	XT, HoT	XT, HoT	ET, HoT	HoT	XT, HoT	XT, HoT	XT, HoT	XT	XT, HoT
Limited horizontal eye movements	−	B	L	B	−	−	B	−	B	B	B
Limited vertical eye movements	B	B	B	B	B	B	B	B	B	B	B
Nystagmus	−	−	−	−	+	−	−	−	−	−	−
Corneal exposure	−	−	−	−	−	−	−	−	+	+	+
*brain malformations (MRI)*											
OBS	−	−	−	−	−	−	−	−	+	+	+
CN3	+	+	+	+	+	+	+	+	+	+	+
CN7: Facial weakness	−	−	−	−	−	−	−	−	+	+	+
EOMs	+	+	+	+	+	+	+	+	+	+	+
Corpus callosum	/	−	−	−	−	−	+	+	+	+	+
Cerebellum	/	−	−	−	−	−	−	+	−	−	+
Anterior commissure	/	−	−	−	−	−	+	+	+	+	+
Basal ganglia	/	−	−	−	−	−	−	+	−	−	+
Internal capsule	/	−	−	−	−	−	+	+	−	−	+
External capsule	/	−	−	−	−	−	−	+	−	−	−
Thalamus	/	−	−	−	−	−	−	+	−	−	+
Lateral ventricle	/	−	−	−	−	−	−	+	+	+	+
Sylvian fissure	/	−	−	−	−	−	−	−	−	−	+
Hippocampus	/	−	−	−	−	−	−	−	−	−	+
*Others*											
Intellectual impairment	−	−	−	−	−	−	−	+	+	+	+
Congenital contractures	−	−	−	−	−	−	−	−	−	−	+
Leg length discrepancy	−	−	−	−	−	−	−	−	−	−	+
Impaired gait	−	−	−	−	−	−	−	+	+	+	+
Cryptorchidism	−	†	−	−	†	†	†	−	+	†	+
Infertility										+*	
Cyclic vomiting	−	−	−	−	−	−	−	−	−	−	+

M, Male; F, Female; y, year; AA, Amino acid; R, Right; B, Bilateral; L, Left; XT, Exotropia; ET, Esotropia; HoT, Hypotropia; OBS, Olfactory bulbs and sulci; EOMs, Extraocular muscles; /, Not available; † Not applicable; + *, Infertility was found in subsequent clinical follow-up

**Fig. 6:**
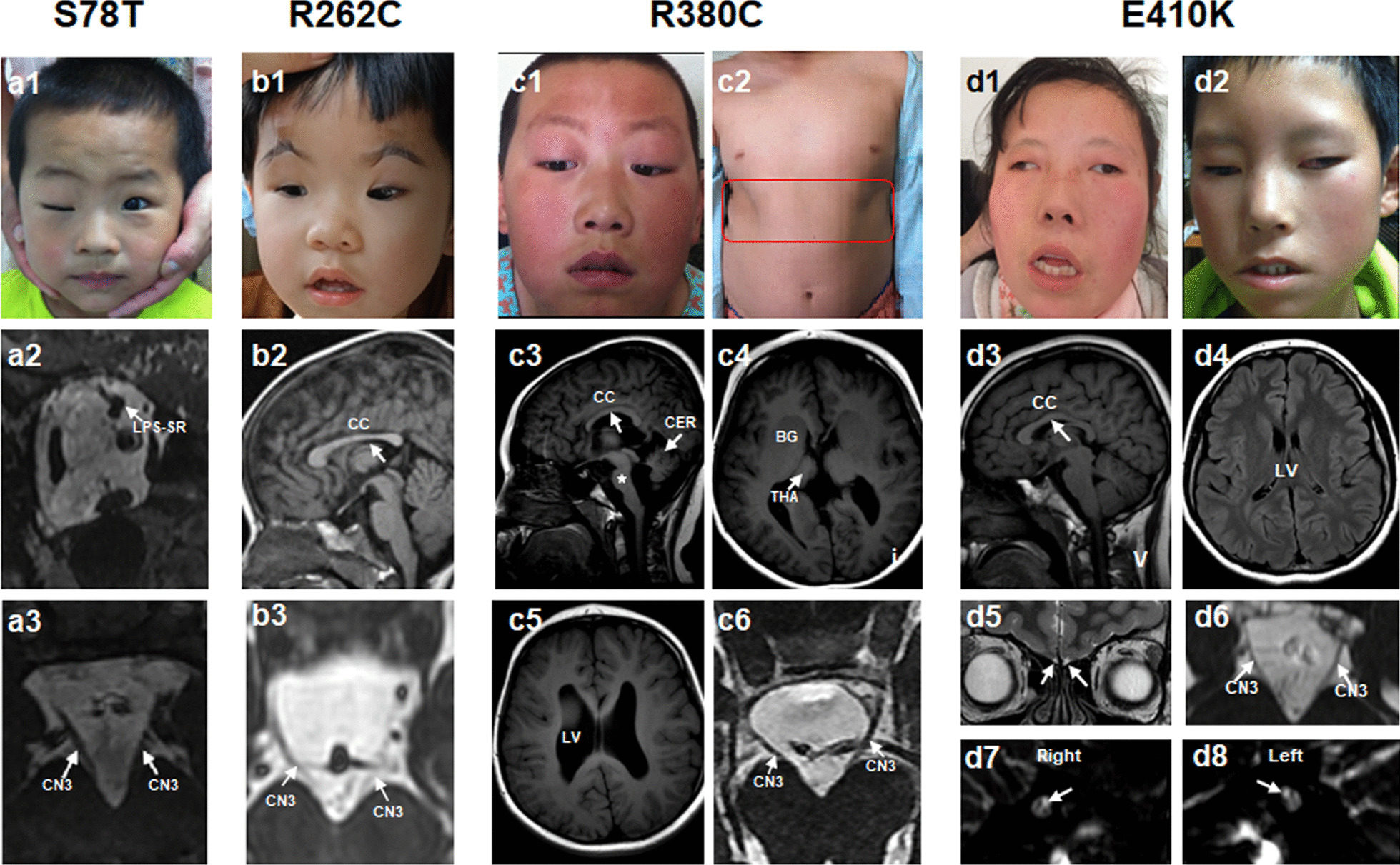
Clinical and MRI spectrum of CFEOM patients with *TUBB3* variants. (**a1**–**a3**), Patient C9-2 harboring S78T variant. **a1** Congenital ptosis of right eye. **a2** Atrophy of levator palpebrae superioris-superior rectus (arrow) in right eye. **a3** Hypoplasia of the CN3 at the brainstem. The MRI was taken at the age of 4y. **b1**–**b3** Patient C65 harboring R262C variant. **b1** Congenital bilateral ptosis. **b2**, **b3** Hypoplasia of CC and CN3. The MRI was taken at the age of 1y. **c1**–**c6** Patient C6 harboring R380C variant. **c1**, **c2** Congenital bilateral ptosis and chest wall malformation (red box). **c3**, **c4** Hypoplasia of CC, brainstem (c, star), cerebellum (CER), basal ganglia (BG), thalamus (THA) and CN3 with the enlargement of lateral ventricle (LV). The MRI was taken at the age of 11y. **d1**–**d8** Patients harboring E410K variants. **d1**, **d2** Facial pictures of two patients (C21 and C32). **d3**–**d8** Patient C32 exhibits hypoplasia of CC (**d3**), asymmetry of LV (**d4**), absence of olfactory bulbs and sulci (**d5**, arrow), hypoplasia of CN3 (**d6**) and absence of bilateral CN7 (**d7**, **d8**, arrow). The MRI was taken at the age of 19y

**Fig. 7:**
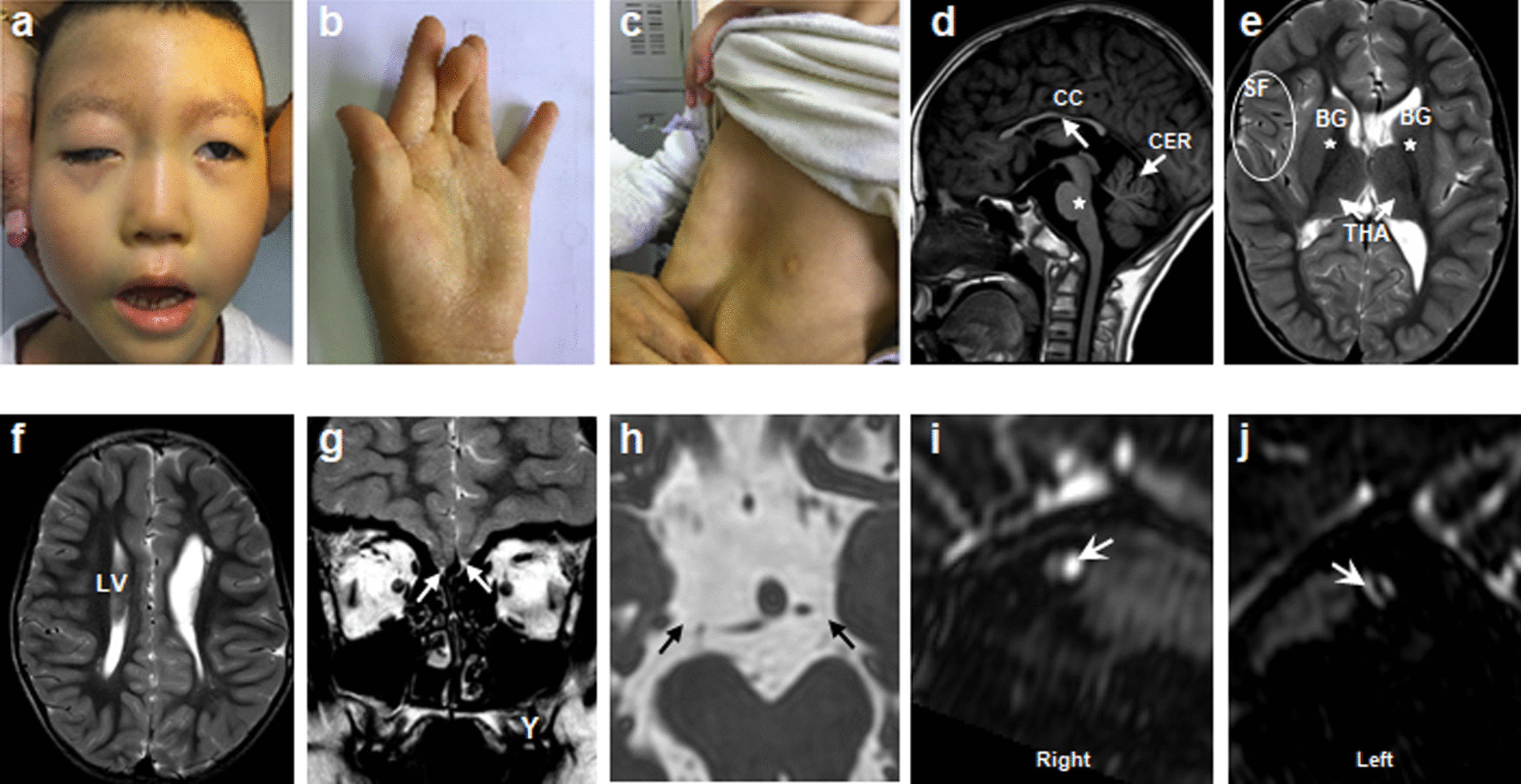
Clinical spectrum and MRI findings of CFEOM patients with *TUBB3* R262H variant. **a**–**c** Congenital bilateral ptosis, facial weakness, joint contracture and funnel chest. **d**–**f** Hypoplasia of CC, brainstem (**d**, star), CER, BG, THA with asymmetry of Sylvian fissure (SF) and LV. **g**–**j** Absence of olfactory bulbs and sulci (**g**, arrow), CN3 (**h**, arrow) and CN7 (**i**, **j**, arrow). The MRI was taken at the age of 4y

The patients harboring R262C and S78T showed milder phenotypes compared to the patients with R380C, E410K and R262H variants. The majority of R262C patients and one S78T patient showed isolated CFEOM, except patient C65 (R262C) who showed the absence of anterior commissure and the hypoplasia of callosum corpus and internal capsule (Fig. 6). All the CFEOM patients with R380C, E410K and R262H variants were accompanied by multiple congenital craniocerebral and systemic malformations (Figs. 6, 7 and Table 3). Facial weakness and hypogonadotropic hypogonadism occurred in the patients harboring E410K and R262H variants. Psychomotor retardation occurred in patients with R380C, E410K and R262H variants. The patient carrying the R380C variant had delayed developmental milestones, and he was given rehabilitation treatment for 3 months at the age of 2. At the age of 3, he could walk with the help of others. When walking, his legs were not straight, and he had impaired gait.

Common features of MRI from patients with R380C, R262H and E410K variants were malformations of the corpus callosum, anterior commissure and lateral ventricle. Malformations of basal ganglia, thalamus, brainstem and cerebellar were correlated with specific variants—R380C and R262H variants. Absence of facial nerves (CN7) and dysplasia of olfactory bulbs and sulci (OBS) were correlated with R262H and E410K variants (Figs. 6, 7).

### DRS

A total number of 11 DRS patients from 8 families (2 familial and 6 sporadic) were enrolled, and they all exhibited congenital esotropia with limited horizontal eye movement accompanied by globe retraction which results in narrowing of the palpebral fissure. MRI revealed hypoplasia of bilateral abducens nerves (CN6) and dysinnervation of CN3.

**Fig. 8:**
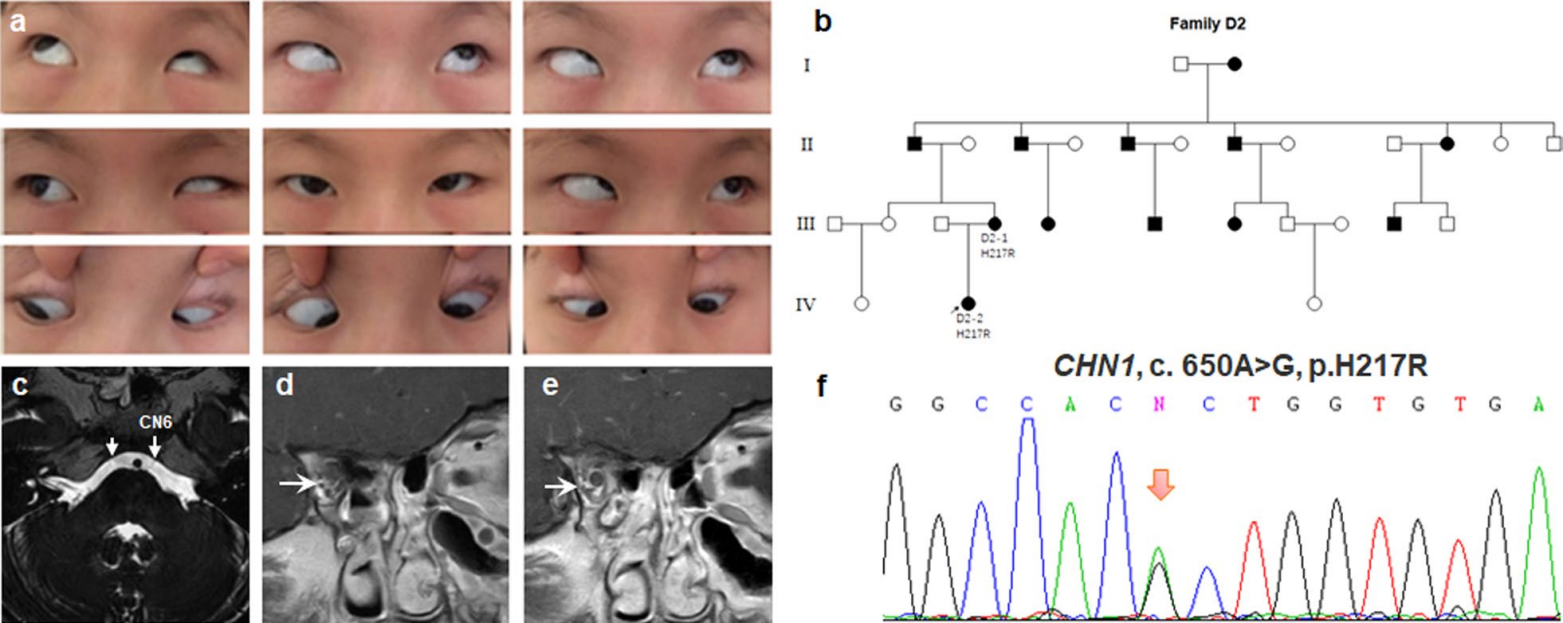
Photographs of the patient D2-1 harboring H217R variant in *CHN1* gene. **a** Nine gaze photographs show limited abduction, globe retraction with narrowing of the palpebral fissure on attempted adduction. **b** Pedigree of autosomal dominant D2. **c**–**e** Hypoplasia of the bilateral CN6 and aberrant innervation of LR by the inferior branches of CN3. **d**, **e** Two consecutive coronal MRI images to show the course of CN3. The MRI was taken at the age of 5y. **f** Genomic sequence chromatogram of heterozygous variant c.650A > G, p.H217R (arrow) in *CHN1* gene

No pathogenic variants were identified in sporadic DRS cases. Only one pedigree (D2) found a novel variant (c. 650A > G, p. H217R) in *CHN1* gene. The patient D2-2 had congenital esotropia, nystagmus, limited horizontal eye movements and globe retraction with narrowing of the palpebral fissure on attempted adduction. The patient had classic DRS and additional vertical movement anomalies, with the right adducted eye slightly depressed and the left adducted eye slightly elevated. MRI revealed hypoplasia of CN6 and aberrant innervation of the lateral rectus (LR) by the inferior branches of CN3 (Fig. 8).

### MBS and HGPPS

**Fig. 9:**
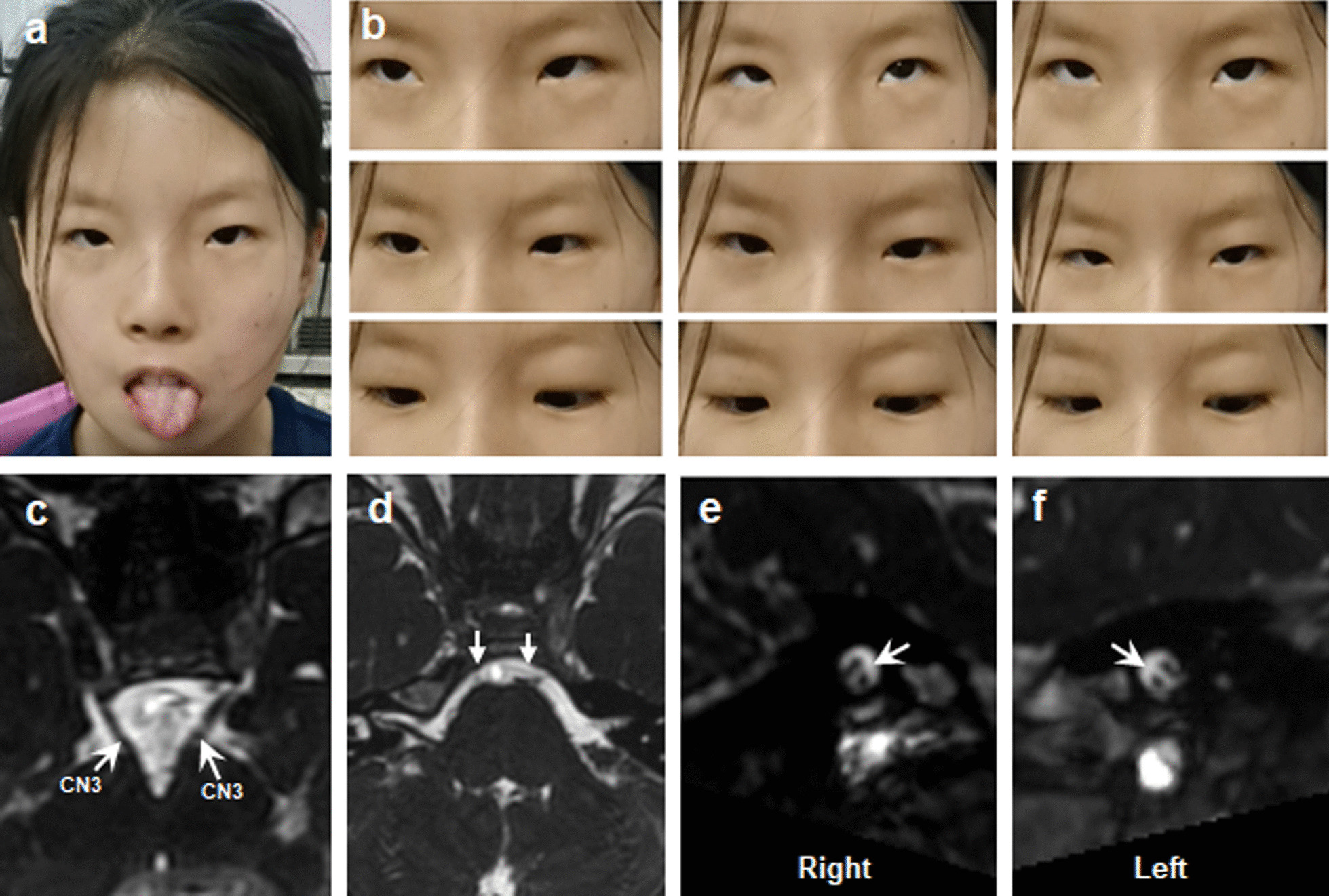
Clinical spectrum and MRI findings of patient M11 with MBS. **a** Facial photograph shows bilateral congenital facial weakness and hypoplasia of tongue. **b** Nine gaze photographs show limited eye movements. **c**–**f** MRI findings of patient C3 (The MRI was taken at the age of 9). Normal CN3 and hypoplasia of the bilateral CN6 and CN7 (**c**–**f**, arrow)

The study enrolled 17 MBS and 5 HGPPS patients. All MBS patients were sporadic and showed congenital, non-progressive abduction deficits and facial weakness. MRI revealed hypoplasia of CN6 and CN7 in all MBS patients (Fig. 9). Notably, nine MBS patients (52.9%, 9/17) had intrauterine exposure to adverse factors.

**Fig. 10:**
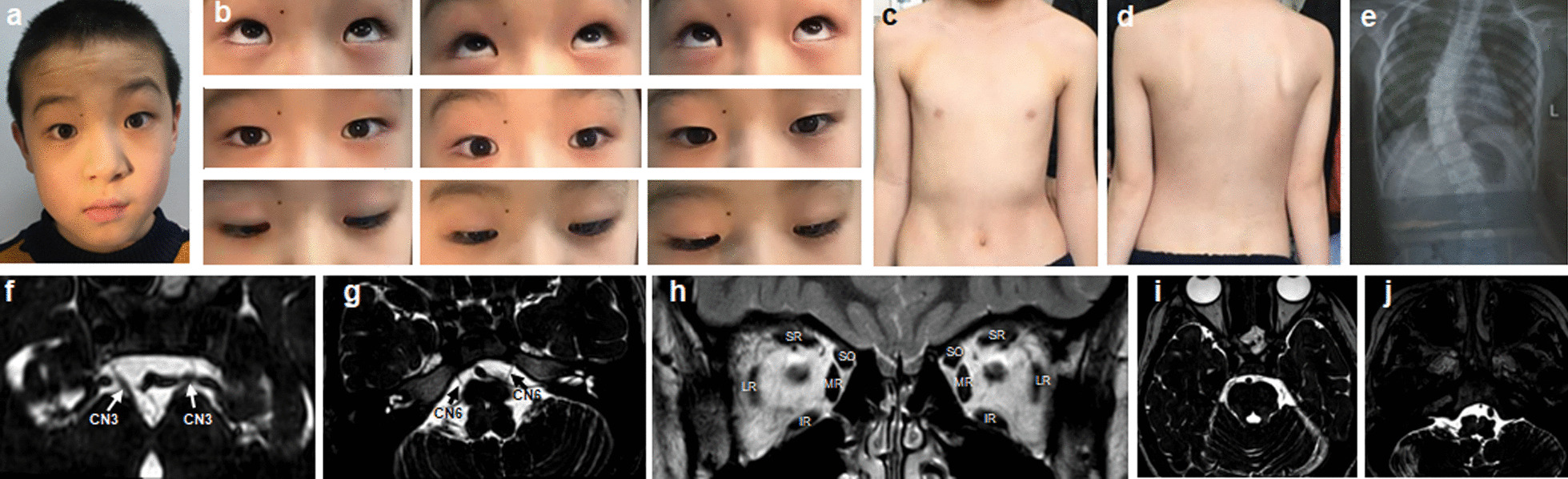
Clinical spectrum, MRI findings and spine X-ray of patient H4 with HGPPS. **a** Facial photograph. **b** Nine gaze photographs show limited horizontal eye movements and full vertical eye movements. **c**, **d** Photographs of the body show obvious scoliosis. **e** Spine X-ray shows obvious thoracolumbar scoliosis. **f**–**h** Normal CN3, CN6 and EOMs. The MRI was taken at the age of 9y. **i** A deep posterior midsagittal cleft of the pons. **j** Flattening and butterfly-like morphology in the medulla

All HGPPS patients were from families of non-consanguineous marriage and showed absence of conjugate horizontal eye movements with intact vertical eye movements, accompanied by variable degrees of scoliosis (Fig. 10). Among four patients who underwent MRI, all had the typical radiographic features of HGPPS, including a deep posterior midsagittal cleft of the pons and butterfly-like morphology in the medulla.

No definite pathogenic variants in known candidate genes were identified in MBS and HGPPS patients.

## Discussion

In this study, we combined MRI and WES to reveal and broaden the phenotypic and genotypic spectrums in 122 Chinese CCDDs patients from 96 families.

### *KIF21A* and *TUBB3* were the common pathogenic genes of Chinese *CFEOM*

The mutation detection rate of CFEOM was the highest (63.6%, 42/66), which was much higher than other disease groups (DRS, MBS and HGPPS). And the mutation detection rate of familial CFEOM was 100%. The common pathogenic genes of CFEOM were *KIF21A* (73.8%, 31/42) and *TUBB3* (26.2%,11/42) genes. Whitman et al. summarized the results of previous researches and found that the variants of *KIF21A*, *TUBB3*, *PHOX2A*, *TUBA1A*, *COL25A1* and *TUBB2B* were identified in 55%, 35%, 10%, < 1%, < 1% and < 1% of the CFEOM patients, respectively [22]. These results are consistent with our current research findings.

KIF21A belongs to a family of kinesin motor protein, which involved in cargo transport along microtubules [5]. Pathogenic variants in *KIF21A* gene may cause CFEOM by modifying the autoinhibitory interaction between the motor domain and a regulatory region in the stalk [23]. The majority of previously reported variants located in coiled coil domains of KIF21A, meanwhile, the CFEOM patients are almost all present with isolated phenotype [5]. However, the novel variant F355S identified in our study was located in conserved N-terminal motor domain, and the patient showed rare syndromic phenotype. To our knowledge, only three variants located in the motor region of KIF21A have been reported, they were C28W, D352E, M356T [5, 24, 25]. The patients harboring C28W and M356T variants showed isolated CFEOM, while the patients harboring D352E variant present with syndromic phenotype. Notably, patient harboring F355S in this study and the patient harboring D352E had the similar phenotypes, including CFEOM, facial weakness, frontal bony prominence, exposure keratitis and delayed developmental milestones. The difference is that the F355S patient also had the hypoplasia of corpus callosum in MRI. We speculate that the F355S may attenuate autoinhibition to a greater degree, or induce disease via different pathways [26].

*TUBB3* gene encodes the neuron-specific beta-tubulin isotype 3 which plays a critical role in axon guidance and neuronal migration. Pathogenic variants in *TUBB3* may cause CFEOM by altering dynamic instability of microtubules or disrupting the interaction of microtubules with kinesin motors [27].

We found a novel variant TUBB3-S78T in a CFEOM pedigree, the proband has normal developmental history (C9-2). The S78T is located in the N-terminal domain of TUBB3, so far, several variants have been reported in the region of S78. Whitman et al. [28] reported the patients harboring G71R and G98S variations presented with syndromic phenotypes, manifested as CFEOM, nystagmus, torticollis, developmental delay, intellectual and social disabilities. MRI showed the hypoplasia of the corpus callosum and anterior commissure, malformations of hippocampi, thalami, basal ganglia and cerebella, and hypoplasia of brainstem and cranial nerve. The patients harboring the S78T variant in this study and the patient harboring R62Q variant reported by Max A. Tischfield in 2010 presented with isolated CFEOM [6]. Although the S78T patient (C9-2) underwent MRI exam of the ocular motor nerves, and had the normal corpus callosum configuration, however the high resolution brain MRI data was absent, so we cannot rule out the possibility that he has subtle cortical dysplasia. In addition, we noted that G82R have been reported to cause MCD without CFEOM [29].

Compared to patients harboring *KIF21A* variants, the CFEOM patients with *TUBB3* variants tend to show syndromic phenotypes. In the past, researchers named the disease caused by the *TUBB3* variants as the “TUBB3 Syndromes” [6]. In recent years, researchers found that different variants of *TUBB3* usually produce different syndromic phenotypes [6]. In our cohort, the patients harboring E410K and R262H variants of *TUBB3* gene exhibited CFEOM and accompanied by different multiple congenital malformations, and their clinical and MRI findings were highly similar to previous reports [4, 30], which confirmed that the correlations between specific variants and phenotypes were strong. Therefore, researchers preferred to name these syndromic phenotypes after specific variants. Up to date, “TUBB3 E410K syndrome”, “TUBB3 R262H syndrome” and “TUBB3 M323V syndrome” were used to name the diseases caused by E410K, R262H and M323V variants, respectively [4, 31, 32]. Furthermore, the patients with “TUBB3 E410K syndrome” and “TUBB3 R262H syndrome” were easily misdiagnosed as MBS owing to similar clinical manifestations (facial weakness and limitation of eye movements etc.) [33]. MRI, WES and specific general examinations based on the good phenotype-genotype correlations can contribute to accurate diagnosis and follow-up.

*TUBB3* R380C variant was identified in one CFEOM patient (C6) in our cohort, and this variant has been previously reported only once in a CFEOM patient [6]. The clinical and MRI findings of these two patients were highly similar, both of them manifested CFEOM with intellectual impairment, multiple congenital craniocerebral malformations which involved in commissural fiber, brainstem, basal ganglia, thalamus, cerebral ventricle and cerebellum etc. However, “bilateral chest wall malformation” and impaired gait were observed only in our R380C patient. Previous research indicated sensorimotor polyneuropathy occurred in individuals harboring R262H, E410K and D417H/N [6]. We speculated that the R380C patient in this study may have similar sensorimotor polyneuropathy, and electromyography results are needed to confirm the suspected diagnosis.

Furthermore, we also noted that *TUBB3* R380C variant had been reported in patients diagnosed with “neurodevelopmental disabilities” and “cerebellar dysplasia (CD)” [34, 35], however the ocular manifestations of the patients were not described clearly by authors.

We noted that Chew et al. and Whitman et al. reported CFEOM patients harboring E410K and R262H variants were accompanied by Kallmann syndrome (anosmia with hypogonadal hypogonadism) [4, 31]. Furthermore, Yasuko Nakamura et al. also reported E410K patients presented with Kallmann syndrome [33].

In present study, three patients with *TUBB3* E410K and R262H variants (C21, C32 and C52) fitted the diagnostic criteria of Kallmann syndrome, confirmed the previous report results and suggested that *TUBB3* gene may involve in the pathogenesis of Kallmann syndrome [4].

### Genetic screening results of sporadic DRS, MBS and HGPPS cases were poor

DRS is a congenital motility disorder which characterized by restricted abduction or adduction and accompanied by globe retraction and narrowing of the palpebral fissure [36]. *CHN1* gene was a common causative gene for familial non-syndromic DRS, which was identified in approximately 35% of familial DRS cases [11]. Functional analysis revealed that *CHN1* variants caused CN6 growth stalled, leading to secondary dysinnervation of the LR by the CN3 [37]. To date, variant detection rate was poor in sporadic isolated DRS patients, so genetic screening is suggestive for those individuals with positive family history [38]. In our DRS cohort (2 familial and 6 sporadic), we identified one novel variant H217R in *CHN1* gene in a large DRS family, no causative genes were identified in all sporadic DRS cases and one DRS family. These results were similar to the previous study [38].

MBS is a rare disease with minimum diagnostic criteria of congenital limitation of ocular abduction and facial weakness [3]. In 2015, Laura Tomas-Roca et al. reported that *PLXND1* and *REV3L* may be pathogenic genes for MBS, and animal researches indicated *PLXND1* and *REV3L* may cause a defect in the facial branchiomotor neuron migration [17]. This was the first report of pathogenic gene for MBS, however, these two genes have not been reported again in other MBS cases to date. In addition to genetic factors, intrauterine environmental factors were also reported be involved in MBS [39]. In our study, no causative gene was identified in all MBS patients, and more than half of the patients had intrauterine exposure to adverse factors. These results suggested that multifactorial pathogenic mechanism maybe exist in MBS.

HGPPS is a rare autosomal recessive disorder characterized by severe restriction of conjugate horizontal eye movements and progressive scoliosis [16]. Reviewed the published reports about HGPPS, it could be found that *ROBO3* was the pathogenic gene and the HGPPS patients harboring *ROBO3* variants often existed parental consanguinity [40, 41]. *ROBO3* plays an important role in axonal guidance which mediate midline crossing of neurons during embryogenesis of the spinal cord [16]. Our study enrolled five sporadic HGPPS patients who were from non-consanguineous family. No homozygous variants of *ROBO3* gene were found, and the possibility of compound heterozygous mutation could not be ruled out. More than a dozen heterozygous variants of *ROBO3* gene were identified in five HGPPS patients, however, no variants were classified as pathogenic or likely pathogenic according to ACMG standards, so the pathogenicity of these variants cannot be determined.

The mutation detection rates in sporadic isolated DRS, MBS and non-consanguineous families with HGPPS were poor in this study. The reasons could be multiple, such as epigenetic factors, environmental or other nongenetic factors may be involved in the pathogenesis of diseases.

The phenotypes of CCDDs are diverse and complex, often involving multiple tissues and organs, many new MRI and clinical phenotypes have been reported and discovered. Face to face communication with radiologist is necessary. At the same time, the participation of clinical multidisciplinary doctors is required to carry out professional examinations, diagnosis and treatment. Considering these facts, future efforts need to focus on mining, refining and quantifying extraocular manifestations to improve our understanding of clinical and genetic basis of CCDDs. Meanwhile, the novel variants need future functional studies to determine its pathogenicity.

## Conclusions

This study was the largest clinical and genetic research for CCDDs patients from China, the results broadened the phenotypic and genotypic spectrums of CCDDs. *KIF21A* and *TUBB3* were the common pathogenic genes of Chinese CFEOM. Compared with patients with *KIF21A* variants, patients with *TUBB3* variants have better genotype–phenotype associations. The patients harboring R380C, E410K and R262H variants in *TUBB3* gene and F355S amino acid variant in *KIF21A* gene exhibited syndromic phenotypes. Genetic screening results of sporadic DRS, MBS and HGPPS cases were poor. These findings were highly consistent with previous results. The combination of MRI and WES was effective for accurate and supportive diagnosis of clinical suspected CCDDs, and the research results would be valuable for future gene therapy and facilitate further understanding of pathogenic mechanism of CCDDs.

## Methods

### Patients

This was a prospective cohort study with retrospective data analysis. A total of 122 self-reported Han patients from 96 not known to be related families with CCDDs were enrolled in the outpatient department of Beijing Tongren Hospital affiliated to Capital Medical University from January 2013 to December 2021.

The inclusion criterion was (1) congenital, non-progressive restrictive eye movements and/or dysplasia of the cranial nerves in MRI, or (2) congenital, non-progressive limitation of horizontal eye movements and progressive scoliosis, with or without dysplasia of the cranial nerves [1].

The study complied with the tenets of the Declaration of Helsinki and was approved by the Institution Review Board of Beijing Tongren Hospital, Beijing, China (TRECKY2018-035). All participants we enrolled gave written informed consent.

### Clinical evaluation and MRI

All participants underwent a detailed ophthalmic and physical examinations. The ophthalmic examinations included cycloplegic refraction, best-corrected visual acuity (BCVA), binocular alignment, eye movements, measurement of the height of palpebral fissures and the function of levator palpebrae muscles, slit lamp examination and fundus examination.

Multi-positional high-resolution MRI was performed in 96 patients from 88 families with a General Discovery MR750 3.0-T Twinspeed scanner. Children under 10 years old were given oral chloral hydrate (10–15 mg/kg) 30 min before examination. Imaging of cranial nerves at the brainstem was conducted with an 8HR brain coil and adopted the three-dimensional fast imaging employing steady—state acquisition (3D-FIESTA) sequence. Parameters were as follows: axial scanning; repetition time (TR), 4.28 ms; echo time (TE), 2.04 ms; flip angle (FA), 60°; the field of view (FOV), 16 × 16 cm; Matrix, 256 × 320; section thickness, 0.8 mm; number of excitation (NEX), 4. Orbital imaging was performed with an 8HR brain coil in oblique coronal planes and applied the following parameters: TR, 325 ms; TE, 9.38 ms; FOV, 17 × 17 cm; matrix, 264 × 256; section thickness, 3.0 mm; layer spacing, 0.3 mm; NEX, 2. Scanning of the brain structure was conducted with a phased array coil in head-cross section and the parameters were as follows: Fast spin-echo/T2-weighted images (FSE/T2WI); section thickness, 6.0 mm; layer spacing, 1.0 mm.

### Whole exome sequencing and bioinformatics analysis

Whole blood samples were collected from all participants and their nuclear family members, genomic DNA was extracted using TianGen Biochemical DNA isolation kit (Beijing Tiangen Biochemical Co., Ltd, Beijing, China).

Whole exome sequencing was performed at Beijing Novogene Bioinformatics Technology Co., Ltd (Beijing, China). Agilent SureSelect Human All ExonV6 kit was used to capture whole exome sequence. Sequencing was performed on illumina HiSeq4000 platform using PE150 sequencing strategy.

Valid sequencing data was mapped to the reference genome (GRCh37/hg19) by BurrowsWheeler Aligner (BWA) software. Samtools and Sambamba were used to sort bam files. Samtools and bcftools were used to do variants calling and identified SNPs and indels. ANNOVAR was used to do annotation for VCF (Variant Call Format) file with a variety of databases, such as dbSNP, 1000 Genome, GnomAD, CADD and HGMD. Gene transcript annotation databases, such as Consensus CDS, RefSeq, Ensemble and UCSC, were also applied for annotation to determine amino acid alternation. Variants were filtered if MAF (minor allele frequency) > 1% in more than one of the four frequency databases (1000 genomic data, esp6500, gnomAD_ALL and gnomAD_EAS). Nonsynonymous variants were screened according to scores of SIFT, Polyphen, VariantTaster and CADD. CNVs analyses were also performed. ACMG guidelines were used to evaluate variants. After the candidate pathogenic variants (*KIF21A, TUBB3, TUBA1A, TUBB2B, TUBB6, PHOX2A, CHN1, MAFB, SALL4, HOXA1, HOXB1, ROBO3, PLXND1, REV3L, COL25A1 and ECEL1*) were found, Sanger sequencing was used for validation. Trios/pedigree analysis were performed to establish co-segregation or de novo status.

## Supplementary Information

**Additional file 1**. Clinical and genetic data of 122 CCDDs patients. Description of data: The table presents detailed data of 122 patients with CCDDs, including the results of ophthalmic and physical examinations, neuroimaging findings and genetic test results.

**Additional file 2**. Allele frequency of three novel variants. Description of data: Three unreported variants were not found in Chinese ethnically-matched databases, gnomAD, etc.

## Data Availability

All data in this study are available upon reasonable request.

## Abbreviations

BCVA: Best-corrected visual acuity
CCDDs: Congenital cranial dysinnervation disorders
CD: Cerebellar dysplasia
CFEOM: Congenital Fibrosis of Extraocular Muscles
CN3: Oculomotor nerves
CN6: Abducens nerves
CN7: Facial nerves
CNVs: Copy number variations
DRS: Duane Retraction Syndrome
EOMs: Extraocular muscles
FA: Flip angle
FOV: The field of view
FSE/T2WI: Fast spin-echo/T2-weighted images
HCFP: Hereditary Congenital Facial Paresis
HGPPS: Horizontal Gaze Palsy with Progressive Scoliosis
LR: Lateral rectus
MBS: Möbius Syndrome
MRI: Magnetic resonance imaging
NEX: Number of excitation
OBS: Olfactory bulbs and sulci
TE: Echo time
TR: Repetition time
WES: Whole exome sequencing
3D-FIESTA: Three-dimensional fast imaging employing steady-state acquisition
